# Reply to Correspondence: Needle Clogging in Biostimulator Injection: Rheological Context and Clinical Experience

**DOI:** 10.1111/jocd.70779

**Published:** 2026-02-26

**Authors:** Flavia Radke, Ulrich Gärtner, Anika Seipp, Marina Gorczyza, Stas Wüst

**Affiliations:** ^1^ Vita Aesthetica Berlin Germany; ^2^ Institute of Anatomy and Cell Biology, Justus Liebig University Giessen Giessen Germany; ^3^ Dermatologie Mahlow, Blankenfelde‐Mahlow Berlin Germany; ^4^ Z282 Medical Affairs Consulting Beverly Massachusetts USA

To the Editor,

We thank the authors of the correspondence in response to our study. While some of the points raised extend beyond the original scope of our investigation, we find it reassuring that the vast majority of our findings are substantiated.

In our original study, we focused on the identification of cannula design features to make them more suitable and safer for the injection of suspension‐based dermal fillers and biostimulators. From our perspective, the main safety concern is the possibility of cannula clogging during the injection, which might lead to increased stress for both the patient and the injector and may compromise patient safety and comfort. Although we agree with the authors of the correspondence that cannula clogging does not occur very frequently in routine practice, we believe that even infrequent events warrant attention and that minimizing the risk is essential to further improving the safety of suspension‐based injections.

The main narrative in the correspondence concerns the contributing factors that would lead to cannula clogging when it is used for suspensions. The authors focus here mostly on the physiochemical properties of the injectable products. We fully agree that suspension‐based products should be prepared at an optimal particle‐to‐medium ratio, which is generally achieved when the products are diluted according to the manufacturer's instructions. We also concur that the manufacturers invest significant effort in optimizing suspension formulations to minimize aggregates and achieve optimal rheological conditions. Whether these optimized suspensions behave as or closely to Newtonian fluids when correctly used remains a matter of debate. However, we agree with the authors that particle size, particle‐to‐medium ratio, viscosity of the medium, and applied pressure are important considerations. Importantly, however, these parameters lie outside of the influence of cannula design.

When focusing purely on the cannula design, we found three main aspects that significantly contribute to increased safety when injecting suspensions. First, the ability of the cannula‐syringe connection to withstand high injection pressures without detachment. Here, we determined that the Luer‐lock mechanism fulfills this criterion. Second, an obstruction‐free inner lumen. As noted by the authors of the correspondence, aggregates might lead to clogging. However, irregularities or obstructions within the lumen may themselves promote aggregation by disturbing flow, causing particles to stall locally, altering the particle‐to‐medium ratio and ultimately leading to clogging of the cannula. Third, the geometry of the exit opening is critical: if the exit opening is smaller than the inner diameter of the cannula, it can literally be the bottleneck leading to clogging, since potential aggregates might pass through the lumen but not fit through the exit opening.

In this context, it is interesting that the authors are talking about only two principles that may cause cannula clogging: the size of the inner diameter of the cannula vs. suspension particle size and the completeness of the reconstitution of the suspension before injection. While this might be true under laboratory settings, in a real‐world clinical use introduces additional variabilities. In routine practice, cannulas are frequently employed using fanning technique, meaning that the cannula will be moved at least 5–10 times back and forth in the subdermal plane. Considering our results, which demonstrate a jagged edge of the TSK brand cannula, we strongly believe that the back‐and‐forth motions of the fanning technique will more likely lead to scraping of connective tissue into the cannula lumen—especially if the product application happens only during one motion, for example, retrograde. Therefore, a smooth edge, as displayed in the Mirror Soft cannula, might be considered a fourth criterion when it comes to cannula optimization for suspension injections.

Finally, the authors discuss the use of sharp hypodermic needles for intradermal applications of suspension‐based fillers. Besides the main differences that sharp needles for such injections are usually much shorter than cannulas and have an exit opening as wide as the lumen diameter, and that the opening is fully aligned with the direction of the flow through the needle lumen—unlike the orthogonal orientation of the exit opening of the cannula, the use of fine gauge hypodermic needles for intradermal delivery falls outside the scope of our investigation, which focused exclusively on cannula design for suspension injection.

## Conflicts of Interest

F.R. is a trainer and consultant for Galderma. M.G. is a consultant for Allergan Aesthetics, Galderma, Merz Aesthetics and PromaMedical. S.W. is a consultant for Galderma, Lemma Health and PromaMedical.

## Data Availability

Data sharing not applicable to this article as no datasets were generated or analyzed during the current study.

